# Spontaneous Spleen Rupture in a Teenager: An Uncommon Cause of Acute Abdomen

**DOI:** 10.1155/2013/675372

**Published:** 2013-04-23

**Authors:** Verroiotou Maria, Al Mogrampi Saad, Ioannis Fardellas

**Affiliations:** Surgical Clinic, Naousa General Hospital, Afoi Lanara & Pexlivanou 3, 59200 Imathia, Greece

## Abstract

Spontaneous spleen rupture is a rare complication of infectious diseases and it can become a potentially life-threatening condition if not diagnosed in time. A 17-year-old Greek female presented to the ER due to acute abdominal pain, mainly of the left upper quadrant. She had no recent report of trauma. The patient was pale, her blood pressure was 90/70 mmHg, and her pulse was 120 b/min. Clinical examination of the abdomen revealed muscle contraction and resistance. The patient was submitted to an ultrasound of the upper abdomen and to a CT scanning of the abdomen that revealed an extended intraperitoneal hemorrhage due to spleen rupture. Due to the patient's hemodynamic instability, she was taken to the operation room and splenectomy was performed. Following a series of laboratory examinations, the patient was diagnosed to be positive for current cytomegalovirus infection. The postoperative course was uneventful, and in a two year follow-up the patient is symptom-free. Spontaneous spleen rupture due to Cytomegalovirus infection is a rare clinical entity, described in few case reports in the world literature and should always be taken into consideration in differential diagnosis of acute abdomen, especially in adolescents with no recent report of trauma.

## 1. Introduction

Spontaneous rupture of the spleen is an extremely rare condition that may be caused by intrinsic or extrinsic factors [1]. According to the international electronic database, there is a short number of case reports that refer to the spontaneous spleen rupture associated with primary CMV infection.

## 2. Case Presentation

A 17-year-old female presented to the ER with acute left upper quadrant abdominal pain. She reported no recent trauma and the only notable item in her medical history was the presence of flu-like symptoms one week before her admission. 

The patient was pale, she had a low-grade fever, blood pressure 90/70 mmHg and pulse rate 120 b/min. Physical examination revealed muscle contraction and resistance during the palpation of the left upper abdomen and the initial blood count showed red blood cells 3.96 × 10^6^/*μ*L, Hb 10.10 g/dL, Hct 32.20%, Rdw 17.30%, platelets 189 × 10^3^/*μ*L, white blood cells 10 × 10^3^/*μ*L, neutrophils 22.5%, and lymphocytes 70.5%, with 12% of the lymphocytes being atypical. The rest of the laboratory exams revealed fibrinogen 1.45 g/L, D-dimer: 742 *μ*g/L, and AST and ALT values were tripled.

**Figure 1:**
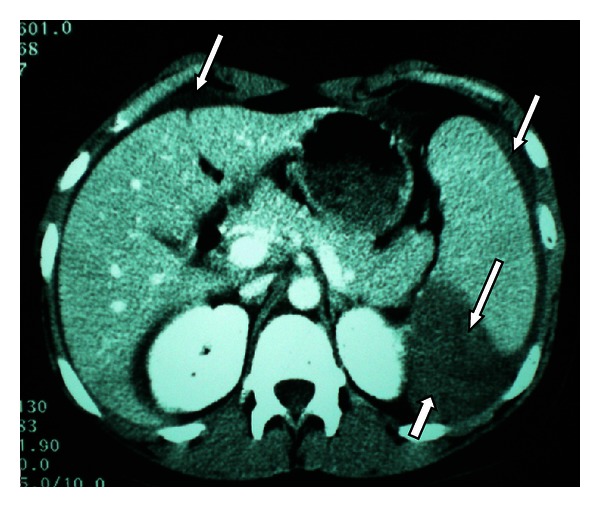
Rupture of the lower pole of the spleen, partial posterior rupture of the spleen, and intraperitoneal hemorrhage.

On this state the patient was treated with crystalloids, while blood transfusion was not considered, having Hb: 10.10 g/dL. An ultrasound of the upper abdomen revealed moderate splenomegaly (12 × 9 cm), with heterogeneity of the lower pole of the spleen and the presence of a small quantity of blood around the spleen and in the Douglas cavity. Since abdomen ultrasound was not considered diagnostic, the patient was submitted to a CT scanning, which confirmed the splenomegaly and the heterogeneity of the spleen and revealed a rupture of the lower pole and a partial posterior rupture, associated with the presence of free fluid throughout the whole peritoneal cavity (Figure 1).

Despite the administration of fluids, the patient remained hemodynamically unstable, with a decrease of hemoglobin to 7 g/dL and therefore she was taken to the surgical chamber. Given the critical situation of the patient and the absence of a confirmed diagnosis, she was submitted to splenectomy and three units of blood were used for transfusion. 

The patient had a normal postoperative course, with a full recovery, and was released the 7th postoperative day. During recovery, the patient was vaccinated against *Streptococcus pneumoniae*, *Neisseria meningitis*, and *Haemophilus influenzae* and was given instructions according to the NHS guidelines for the protection of patients with an absent or dysfunctional spleen [2].

Primary cytomegalovirus infection was suspected by a serological pattern of positive IgM and IgG anti-CMV antibodies, with the following antibody titers: IgM anti-CMV > 14 and IgG anti-CMV > 250, while in the subsequent tests it was noticed that there are a decrease of IgM antibodies (IgM anti-CMV > 8) and a persistence of IgG antibodies. Mono spot test for EBV was negative. Tests for antibodies against HIV and viral hepatitis A, B, C were and remained negative.

**Figure 2:**
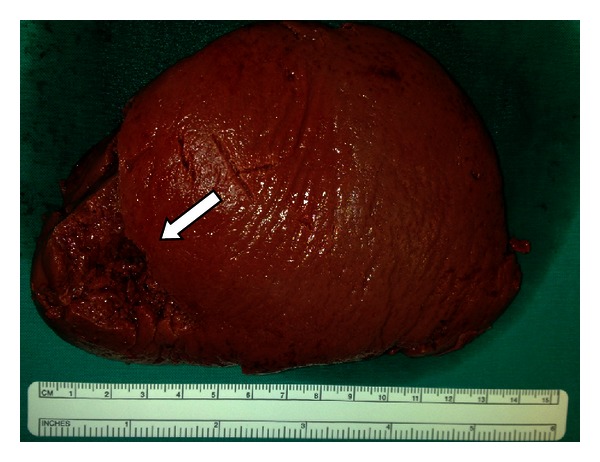
Rupture in the external surface of spleen.

The removed spleen weighed 240 gr., having dimensions 12 × 9 × 5 cm, and according to histopathologic examination, it presented rupture in the external surface (area  5.5 × 2.2 cm) and rupture in the spleen portal (area 5 × 1 cm) (Figure 2). 

**Figure 3:**
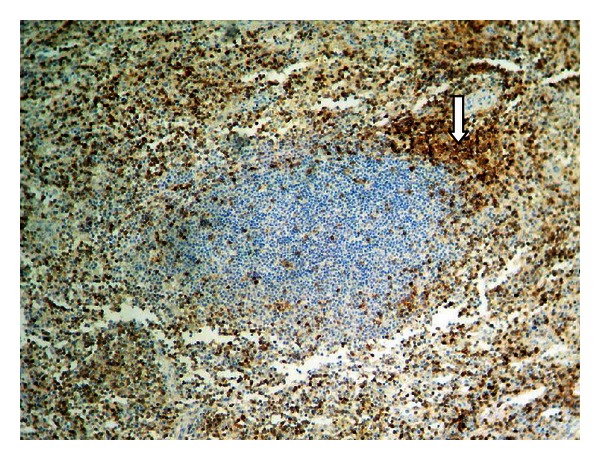
CD3+ ×100. T-cell zone hyperplasia.

**Figure 4:**
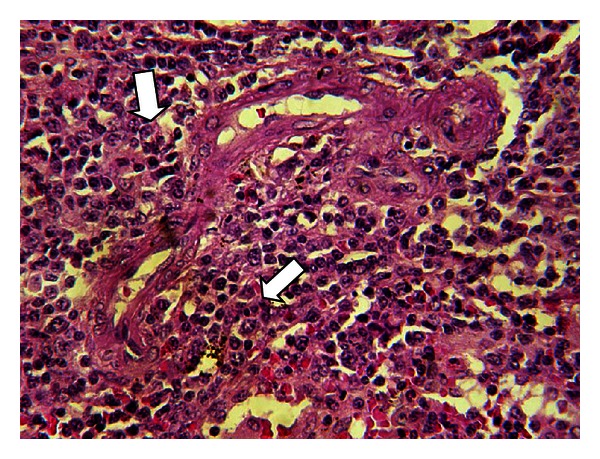
H + E ×400. Splenic parenchyma T-cell proliferation.

The spleen biopsy revealed hemorrhagic impregnation in the areas of rupture and sinusoidal dilatation and hyperemia. The rest of the spleen sections were characterized by T-lymphocyte hyperplasia (Figure 3), but there was no success in identifying CMV inclusions by splenic histopathological findings (Figure 4). 

After the patient was released from the hospital, she was sent to a hematologist and to an internist for further examination and evaluation in order to search for hematological diseases and other pathological conditions that could have been predisposing factors for the splenomegaly and the following spontaneous rupture. The hematologist and the internist completed a numerous series of exams and all diseases that could be responsible for immunodeficiency in the patient were ruled out, including myeloproliferative disorders. Since there had not been identified any other cause for splenomegaly and subsequent spontaneous splenic rupture and given the positive serologic findings for CMV infection, the diagnosis for CMV infection was posed. 

In a two year follow-up, the patient is found to be clinically well and free of symptoms.

## 3. Discussion

Spontaneous spleen rupture is a rare complication of various diseases. According to Renzulli et al. [3], this condition can be classified into atraumatic-idiopathic (7%) and atraumatic pathological splenic rupture (93%). The afore mentioned study identifies six aetiological groups for spontaneous splenic rupture and these groups are in order of frequency as follows: neoplastic (30.3%), infectious (27.3%), inflammatory, noninfectious (20%), drug- and treatment-related (9.2%) and mechanical disorders (6.8%), and normal spleen (6.4%) [3]. Other studies of spontaneous spleen rupture include mainly case reports and refer to splenic infractions, coagulation disorders, thrombocytopenia, portal hypertension, vasculitis, venous thrombosis of the spleen, and focal splenic lesions [1].

Infectious diseases can induce a swelling of the spleen due to hyperplasia of the pulpa and hyperaemia of the sinus and mantle plexus [1]. According to Alliot et al. [4], splenic fracture, subcapsular hematoma, and frank rupture can be described as events along a single continuum and therefore the term “splenic rupture” may be misleading. 

In order to identify the frequency of spontaneous splenic rupture associated to CMV infection and to clarify management guidelines, we conducted a PubMed search using the key words “cytomegalovirus” and “spontaneous spleen rupture” and reviewed the series of primary CMV infection in immunocompetent patients published in English and in Greek. We found seven cases of spontaneous spleen rupture in primary CMV infection on the international electronic database and none on the Greek electronic database. It is however likely that many cases of spleen rupture in CMV infection are unreported or misdiagnosed. 

Two cases were excluded because of the comorbidity that was present in these patients before CMV infection. One of them had a pyruvate kinase deficiency and the other had a profound iron deficiency anemia, both patients were treated conservatively [5]. 

In the five remaining cases [4, 6–9], all patients were adults with no history of a disease or a predisposing factor for splenomegaly and spontaneous splenic rupture. 

In all patients, serology examinations were positive for IgM anti-CMV antibodies [4, 6–9], and in one case typical intranuclear inclusions for CMV were identified in the surgical specimen [7]. All patients were treated with splenectomy and had a full recovery [4, 6–9]. 

In our study, the patient was a female adolescent, who was diagnosed with CMV infection due to the positivity of serologic tests. Surgical treatment was decided due to the patient's hemodynamic instability and postoperative period was uneventful.

Spontaneous spleen rupture, associated with CMV infection, should always be suspected in immunocompetent patients, especially in young ones, who present with pain on the left upper abdomen, without a history of trauma and in association with signs of poor specificity. According to Rapp et al., in case of spontaneous splenic rupture, splenectomy is the treatment of choice in patients, hemodynamically unstable with uncontrollable rupture, or recurrent splenic bleeding, while a conservative treatment should be considered in selected, closely monitored patients [10]. 

In conclusion, however rare as it may be as a complication, spontaneous spleen rupture in CMV infection is a potentially life-threatening condition that should be treated according to the hemodynamic condition of the patient and should always be taken into consideration in differential diagnosis of acute abdomen.
